# Effect of Salt Stress on the Phenolic Compounds, Antioxidant Capacity, Microbial Load, and In Vitro Bioaccessibility of Two Microalgae Species (*Phaeodactylum tricornutum* and *Spirulina platensis*)

**DOI:** 10.3390/foods12173185

**Published:** 2023-08-24

**Authors:** Turkan Uzlasir, Serkan Selli, Hasim Kelebek

**Affiliations:** 1 Department of Food Engineering, Faculty of Engineering, Adana Alparslan Turkes Science and Technology University, Adana 01250, Türkiye; uzlasirturkan@gmail.com; 2 Department of Food Engineering, Faculty of Agriculture, Cukurova University, Adana 01330, Türkiye; sselli@cu.edu.tr

**Keywords:** *Phaeodactylum tricornutum*, *Spirulina platensis*, salt stress, phenolics, antioxidant capacity, in vitro digestion

## Abstract

Microalgae have gained attention as alternative food sources due to their nutritional value and biological effects. This study investigated the effect of salt stress on the antioxidant activity, phenolic profile, bioavailability of bioactive compounds, and microbial counts in the blue-green algae *Spirulina platensis* and diatom species *Phaeodactylum tricornutum*. These microalgae were cultured in growth mediums with different salt concentrations (15–35‰) We observed the highest antioxidant activity and phenolic compounds in the control groups. *S. platensis* (20‰) exhibited higher antioxidant activity compared to *P. tricornutum* (30‰), which decreased with increasing salt stress. Using HPLC-DAD-ESI-MS/MS, we identified and quantified 20 and 24 phenolic compounds in the *P. tricornutum* and *S. platensis* culture samples, respectively. The bioavailability of these compounds was assessed through in vitro digestion with the highest amounts observed in the intestinal phase. Salt stress negatively affected the synthesis of bioactive substances. Microbial counts ranged from 300 to 2.78 × 10^4^ cfu/g for the total aerobic mesophilic bacteria and from 10 to 1.35 × 10^4^ cfu/g for yeast/mold in *P. tricornutum* samples while the *S. platensis* samples had microbial counts from 300 to 1.9 × 10^4^ cfu/g and the total aerobic mesophilic bacteria from 10 to 10^4^ cfu/g, respectively. This study suggests that adding salt at different ratios to the nutrient media during the production of *P. tricornutum* and *S. platensis* can impact phenolic compounds, antioxidant capacity, microbial load evaluation, and in vitro bioaccessibility of the studied microalgae.

## 1. Introduction

Microalgae, regarded as one of the earliest photosynthetic organisms on Earth, have existed for 3.5 billion years and stand out as the sole algal group exhibiting a prokaryotic structure among their counterparts [1]. Diatoms (*Bacillariophyceae*) and green algae (*Chlorophyceae*) are known as the most important microalgae groups in terms of their abundance in nature [2]. Diatoms are microscopic unicellular or filamentous algae that have acid and heat-resistant silica shells and are available in marine and freshwater ecosystems as well as in soil and even on moist surfaces [2]. *Phaeodactylum tricornutum* (*P. tricornutum*) is a single-celled eukaryotic diatom belonging to the Pennateae group and is often used as a model organism because of its genome sequence and ease of culturing [2]. It has a brown chromatophore and a large Golgi apparatus in the center of its cell. *Spirulina platensis* (*S. platensis*), on the other hand, is a very important natural food source that has been used since ancient times and has attracted great interest from researchers in recent years due to its high micro and macronutrient contents. It is a prokaryotic blue-green alga with a diameter of about 0.1 mm and grows naturally in the alkaline waters of lakes in warm regions. It is defined as prokaryotic due to the absence of mitochondria, nucleus, Golgi body, endoplasmic reticulum, and vacuoles and is also considered similar to bacteria because of having a similar cell wall [3].

Many phenolic compounds are responsible for the antioxidant activity in the structure of microalgae. These compounds play a significant role in various physiological processes, including stress response allowing the organism to adapt and survive by interacting with its environment. Microalgae are considered natural sources of these bioactive metabolites [4]. In previous studies, various phenolic compounds including protocatechuic acid, catechin, vanillic acid, gallic acid, epicatechin, caffeic acid, coumaric acid, chlorogenic acid, and ferulic acid have been detected in *P. tricornutum* and *S. platensis* [4,5,6,7]. It is known that these bioactive compounds have antioxidant properties as well as beneficial effects by regulating the anticancer, antiviral, antimicrobial, anti-inflammatory, antitumor, and immune systems [6].

It is known that the structures and activities of the bioactive compounds are closely related to the processes in the digestive system. In vitro models have been developed to investigate the effects of digestion on these compounds and predict their bioavailability and release from the food matrix. Bioavailability encompasses the fraction of digested nutrients and bioactive compounds that enter the systemic circulation and is eventually utilized by the body, incorporating bioaccessibility, which quantifies the release of the compound from the matrix within the gastrointestinal tract. Bioactive components such as secondary metabolites are responsible for antioxidant activity and cell protection instead of providing energy to the body [8]. *P. tricornutum* is used in numerous applications in the food, pharmaceutical, cosmetics, and biofuel industries [9] while *S. platensis* is consumed as a food supplement, promoted as a ‘super food’ and sold as capsules, flakes, or dried powder after dehydration by spray drying, freeze drying, sun drying and hot air drying [6].

The biomass productivity of microalgae is significantly influenced by salt concentration, which stands as a crucial environmental factor constraining their growth and impacting their biochemical composition. High salinity levels in plants and microalgae cause ionic, osmotic, and oxidative stress. Microalgae produce various reactive oxygen species (ROS) under salt stress including hydroxyl radicals, hydrogen peroxide, and singlet oxygen [10]. ROS act as secondary messengers in intracellular signaling channels that trigger various abiotic and biotic stress adaptive responses. However, high ROS accumulation is thought to damage macro and micromolecules affecting physiological performance and cellular metabolism [10].

*P. tricornutum*, which has a ciliated cell wall, and *S. platensis*, which has an 86% digestible cell wall are the two most commonly and commercially grown algae species. The phenolic compounds, antioxidant capacity, and in vitro bioaccessibility of these algae species are affected by various factors including the salt concentration of the growth medium. There has been no study in the literature on the effect of salt stress on the antioxidant activity, phenolic profile, and bioaccessibility of the bioactive compounds of *P. tricornutum* and *S. platensis* cultures. Hence, this study focused on the investigation of the effects of different salt concentrations on the antioxidant activity, total aerobic mesophilic bacteria (TAMB), yeast–mold count, phenolic profile, and antioxidant activity of the in vitro digestion of the *P. tricornutum* and *S. platensis* cultures.

## 2. Materials and Methods

### 2.1. P. tricornutum and S. platensis Cultures

*P. tricornutum* and *S. platensis* cultures were grown under controlled laboratory conditions in the Algal Biotechnology Laboratory of the Faculty of Fisheries of Cukurova University, Adana, Turkiye. Zarrouk medium [11] and Si-Walne medium [12] were modified and used for the production of *P. tricornutum* and *S. platensis* cultures, respectively. Sea water was utilized for *P. tricornutum* and pure water was used for *S. platensis*. The cultures were conditioned at room temperature (20 and 25 °C) and grown at a light intensity of 80 μmol photon m^−2^s^−1^ under laboratory conditions. Continuous illumination was applied and the light intensity was checked by a light meter (Licor, LI-250). Fluorescent lamps (Tekfen, TLD, 36 Watt, Istanbul, Turkiye) were utilized and the cultures placed on the shelves were ventilated by an aquarium air pump (3.5 L/min, 5 Watt). The trial culture groups were kep-liter flasks.

The salt concentrations and sample coding used for the *P. tricornutum* cultures were as follows: P15 (‰15), P25 (‰25), P30-C (‰30 control), and P35 (‰35) while for the *S. platensis* cultures, S20-C (‰20 control), S25 (‰25), S30 (‰30) and S35 (‰35) salt concentrations and coding were utilized. These values were determined based on the minimum and maximum salt concentrations at which both species can grow. The optimal concentrations were 30‰ and 20‰ for the *P. tricornutum* and *S. platensis* cultures and these values were used for the control groups in the study. Harvesting was carried out when the growth entered the stationary phase. The sampled biomasses were freeze-dried after the harvest (Teknosem, TRS 4/4V, Istanbul, Turkiye). A vacuum pressure of 0.037 mbar and a temperature of −56 °C were applied during freeze drying without damaging the molecular and physical structure of the sample for about 60 h based on the user’s guide of the dryer. The final water content of the samples was about 8% [13].

### 2.2. Chemicals

Chemicals and standards used in the culture media of *P. tricornutum* and *S. platensis* [catechin (154-23-4), quinic acid (77-95-2), cinnamic acid (140-10-3), caffeic acid (331-39-) 5), vanillic acid (121-34-6), kaempferol (520-18-3), epicatechin (490-46-0), gallic acid (149-91-7), lutein (127-40-2), ferulic acid (1135-24-6), quercetin (849061-97-8) and (±)-6-Hydroxy-2,5,7,8-tetramethylchromane-2-carboxylic acid (Trolox) (53188-07-1)] were purchased from the Sigma company (St. Louis, MO, USA). Acetonitrile (75-05-8), formic acid (64-18-6), 2,2′-azino-bis-(3-ethyl-benzothiazoline-6-sulfonic acid) diammonium salt (ABTS) (30931-67- 0), 2,2-diphenyl-1-picryl hydrazyl (DPPH) (1898-66-4), copper(II) chloride dihydrate (CuCl_2_.2H_2_O) (10125-13-0), 2,9-dimethyl-1,10-phenanthroline (Neocuproine) (484-11-7) were obtained from the Merck company (Gernsheim, Germany). Ultrapure water was obtained by a purifier (Millipore Co., Saint-Quentin, France) and used to prepare the mobile phases in the HPLC analyses. All standards were prepared daily in the analyses.

### 2.3. Extraction of P. tricornutum and S. platensis

Extractions were prepared based on the method available in Kelebek and Selli [14] with some modifications. A sample of 1 g of the freeze-dried samples was mixed with 10 mL of methanol/water (80/20) and kept in an ultrasonic water bath for 3.5 h at a temperature not exceeding 25 °C. Then, the samples were extracted after keeping them in a magnetic stirrer for one night. The extracts were centrifuged at 6500 rpm at 4 °C (Hettich Universal 320R) and the upper clear parts were taken and passed through 0.45 µm filters and stored at 4 °C until the analyses.

### 2.4. Antioxidant Capacity Analyses

The antioxidant capacities of the freeze-dried *P. tricornutum* and *S. platensis* culture samples were determined using three different methods (DPPH, ABTS, and CUPRAC).

#### 2.4.1. DPPH Method

This analysis was performed using 1,1-diphenyl-2-picrylhydrazyl (DPPH), which determined the sample’s ability to inhibit free radicals using a UV-Vis spectrophotometer at 515 nm (BMG Labtech, Spectrostar Nano, Ortenberg, Germany) according to the method outlined by Brand-Williams et al. [15]. By mixing extracts with a DPPH solution, the color of the solution changed from purple to yellow based on the corresponding hydrazine. To determine the reducing ability of the antioxidants towards DPPH, the decrease in absorbance at 515 nm was monitored. Trolox concentrations ranging from 50 to 500 mM were utilized for the calibration and the results were expressed as micromoles of Trolox equivalent (TE) per 100 g of dry weight (mM of TE/100 g of DW).

#### 2.4.2. ABTS Method

In this method, 2,2′-azino-bis 3-ethylbenzothiazoline-6-sulfonic acid (ABTS) was used based on the method of Saafi et al. [16]. Then, 7 mM ABTS was mixed with 2.45 mM potassium bisulfate and kept in the dark for 12–16 h and this solution was diluted with sodium acetate (pH 4.5) buffer to obtain an absorption value of 0.70 ± 0.01 at 734 nm in a spectrophotometer. Then, 2.98 mL of the prepared solution was mixed with 20 μL of sample extract and the absorbance was measured 10 min later in a UV-Vis spectrophotometer (BMG Labtech, Spectrostar Nano, Ortenberg, Germany) at a wavelength of 734 nm. The absorbance values were calculated with the Trolox standard curve and the results were expressed in mM Trolox/100 g DW.

#### 2.4.3. Cupric Reducing Antioxidant Capacity (CUPRAC) Method

The CUPRAC analyses were conducted based on the method described by Apak et al. [17]. The experimental procedure involved the preparation of a 1.0 × 10^−2^ M solution of copper (II) chloride (CuCl_2_.2H_2_O), a 1 M Ammonium acetate buffer at pH 7.0, and a 7.5 × 10^−3^ M solution of neocuproine (2,9-dimethyl-1,10-phenanthroline). A Trolox antioxidant compound stock solution was also prepared at a concentration of 1.0 × 10^−3^ M. In a glass tube, 1 mL of copper (II) solution, neocuproine solution, and ammonium acetate buffer were added sequentially. Then, 0.5 mL of the antioxidant solution and (1.1-x) mL of distilled water were added to the tubes, which were thoroughly shaken. The resulting solutions with a total volume of 4.1 mL were kept at room temperature for 30 min. Subsequently, the absorbance values were measured in a UV-Vis spectrophotometer (BMG Labtech, Spectrostar Nano, Ortenberg, Germany) at 450 nm against the reference solution. The absorbance values were calculated using the standard calibration curve of Trolox (1.22 × 10^−5^, 2.44 × 10^−5^, 3.66 × 10^−5^, 4.88 × 10^−5^, 6.10 × 10^−5^) and expressed as mM Trolox/100 g DW.

### 2.5. Total Phenolic Compounds (TPC) Analysis

TPC analysis was performed using the Folin–Ciocalteu reagent according to the method specified by Shahidi [18]. Then, 200 µL of the extract/standard solution and 1.5 mL of Folin–Ciocalteu reagent (1:10) were added to the spectrophotometer cuvette. After five minutes, 1.5 mL of 6% sodium carbonate solution was added to the tubes and kept for 90 min at room temperature in the dark. The absorbance values were measured at 765 nm in a UV-Vis spectrophotometer. For the calibration curve, a 500 ppm gallic acid solution was prepared and the results were reported as mg/100 g DW.

### 2.6. Analysis of the Phenolic Compounds by LC-ESI-MS/MS

An HPLC system (Agilent Technologies, model 1100) controlled by Windows NT 4.0-based ChemStation software was utilized in the analyses of the phenolic compounds. The HPLC setup included an autosampler (G1367 E, 1260 HIP ALS), a binary pump (G1312 B, 1260 Bin pump), a degasser (G1322 A, 1260 Degasser), and a diode array detector (G1351D 1260 DAD VL). A reversed-phase C18 column (Phenomenex Luna, 4.6 mm × 250 mm, 5 μm particle diameter) was used in the analyses. The mobile phase consisted of two solvents: Solvent A: a mixture of water and formic acid (99:1, *v*/*v*), and Solvent B: a mixture of Solvent A and acetonitrile (60:40, *v*/*v*). Phenolic compounds were eluted under the following conditions: setting to 0.5 mL/min flow rate at 25 °C; isocratic conditions from 0 to 5 min with 5% B; gradient conditions for the following steps: from 5% to 15% B in 5 min; from 15% to 20% B in 15 min; from 20% to 25% B in 10 min; from 25% to 40% B in 15 min; and from 40% to 55% B in 15 min; hold at 55% B in 5 min, from 55% to 70% B in 25 min, from 70% to 100% B in 1 min, hold at 100% in 9 min, from 100% to %5 B in 1 min, from 5% B in 4 min followed by washing and reconditioning of the column. The flow rate was set at 0.5 mL/min while the temperature was maintained at 25 °C. UV-visible spectra from 200 nm to 800 nm were recorded for all peaks [19]. Each compound was identified and assigned by comparing its retention times and UV spectra to authentic standards. The compounds were also analyzed using an Agilent 6430 LC-MS/MS spectrometer with an electrospray ionization (ESI) source. The ESI mass spectrometry (ESI-MS) detection was carried out in negative ion mode under optimized conditions. Quantification of the compounds was achieved using the external standard method with authentic standards. The phenolic contents were calculated based on the method available by Sonmezdag et al. [20]. The calibration curves of the standard phenolic compounds were used to quantify each phenolic compound (compound names and their CAS numbers were given in the chemicals section). Since it was impossible to provide a standard substance for all compounds, calibration curves prepared with structurally-comparable chemicals were used to quantify these compounds. Limits of detection (LOD) and quantification (LOQ) under the current chromatographic conditions were determined with signal-to-noise ratios (S/N) of approximately 3 and 10, respectively. The standard curves were generated using commercial standards at concentrations typically found in microalgae samples (around 1–100 mg/L) with R^2^ values above 0.995. The measurements were performed with three repetitions [19,20].

### 2.7. Detection of the Bioaccessibility of the Bioactive Compounds by In Vitro Digestion

The in vitro digestion method described by Brodkorb et al. [21] was utilized in the study. The method consisted of several steps to simulate the human digestive process. Firstly, a simulated salivary fluid (SSF) was prepared and a sample was mixed with SSF (1:1, *w*/*w*). Next, salivary amylase (75 U/mL) was added and incubated at 37 °C for 2 min to mimic the conditions in the oral phase. Then, a simulated gastric fluid (SGF) was prepared by adjusting the pH of a buffer solution to 2.0 using hydrochloric acid (HCl). Then, the food sample was mixed with the SGF and incubated at 37 °C for a specified period to mimic the conditions in the stomach. After the gastric digestion step, a simulated intestinal fluid (SIF) was prepared by adjusting the pH of a buffer solution to 7.5 using sodium hydroxide (NaOH). The partially digested sample from the gastric digestion step was then mixed with the SIF and further incubated at 37 °C to simulate the conditions in the small intestine. During the digestion process, enzymes such as pepsin (2.000 U/mL) and pancreatin (trypsin activity 100 U/mL) were added to the SGF and SIF, respectively, to mimic the enzymatic activity in the stomach and small intestine. The concentrations of these enzymes were determined based on the physiological levels found in the human digestive system. To monitor the progress of the digestion, samples were collected at specific time intervals during the gastric and intestinal digestion steps. The total phenolic content, antioxidant capacity (DPPH, ABTS, and CUPRAC), and total phenolic substance samples collected from the oral, gastric and intestinal phases were determined with three replicates.

### 2.8. Microbiological Analyses

Samples were taken in stomacher bags in a volume of 10 mL on the days specified for microbiological analysis and 90 mL of 0.1% peptone water was added and homogenized (Masticator IUL). Dilutions were made from the homogenized 10^−1^ dilutions to 10^−8^ dilutions. For microbiological analyses, the samples in the desired dilutions were cultured in two parallel sections and the results obtained were given in cfu/g and the following two analyses were performed for the microbiological analysis of the samples.

Total aerobic mesophilic bacteria (TAMB) count: Colonies formed after 48–72 h of incubation at 30 °C during storage were counted by plating onto plate count agar (PCA) with the pour plate method [22].

Yeast–mold count: Yeast and mold counts were determined by the spreading plate counting method by inoculating onto YGC (Yeast Extract Glucose Chloramphenicol) agar. At the end of 3–5 days of incubation at 30 °C, the colonies were counted and the suspicious ones from the colonies were determined by microscope examination [22].

### 2.9. Statistical Data Analysis

The results obtained in the study were compared with the international literature and subjected to one-way ANOVA analysis using the SPSS statistics program (version:22, SPSS Inc., Chicago, IL, USA). The differences between the means were compared using Duncan’s comparison tests. In addition, the correlation matrix (Pearson correlation coefficients, r) and correlation maps were prepared and examined in the XLSTAT 2019 software (version 2108) to evaluate multiple paired comparisons between the applied salt concentrations in the trials and the antioxidant activity and phenolic compounds.

## 3. Results and Discussions

### 3.1. Antioxidant Capacity Analysis Results

#### 3.1.1. DPPH Method Results

**Figure 1:**
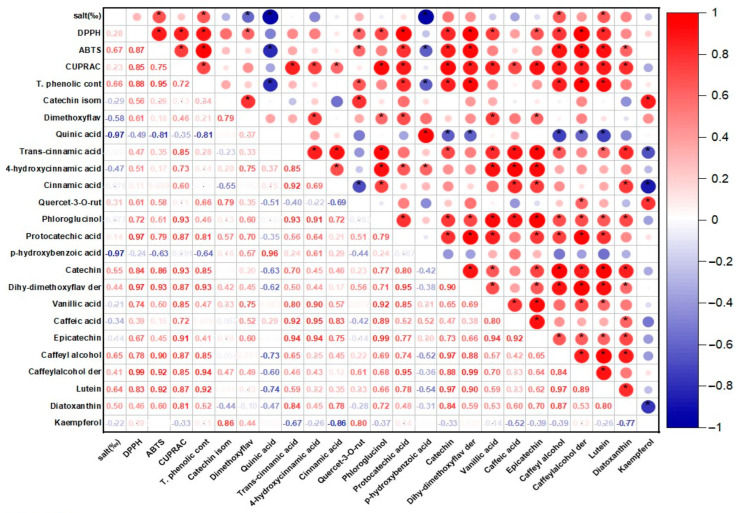
The correlation matrix of the antioxidant activity and phenolic profile of the *P. tricornutum* samples. * *p* ≤ 0.05.

**Figure 2:**
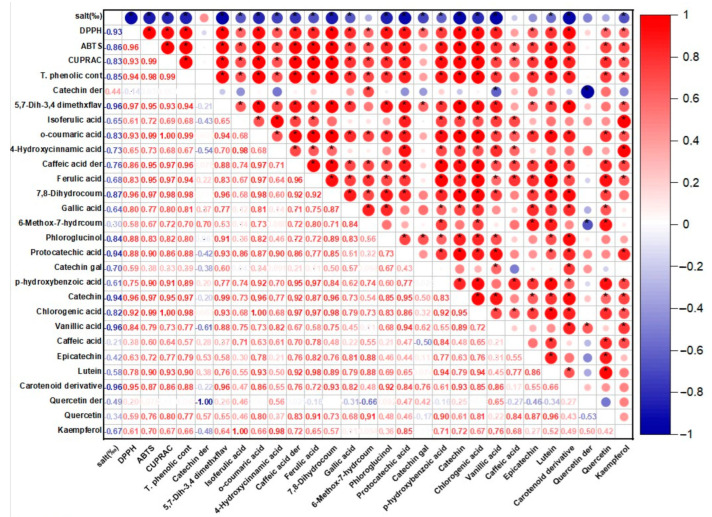
The correlation matrix of the antioxidant activity and phenolic profile of the *S. platensis* samples. * *p* ≤ 0.05.

It is known that phycocyanin, fucoxanthin, and phenolic compounds in the structure of *P. tricornutum* and *S. platensis* samples exhibit antioxidant activity. Table 1 shows the antioxidant capabilities of the samples evaluated in the current study. Statistically significant differences were determined between the DPPH capacities of the samples (*p* < 0.05). In the *P. tricornutum* samples, the highest DPPH capacity was observed in the P30-C sample with 79.40 mM Trolox/100 g DW while the lowest activity was detected in the P25 sample. The highest DPPH capacity in the *S. platensis* samples was observed in the S20-C sample with 172.67 mM Trolox/100 g DW. A positive and moderate correlation (*r* = 0.28) was observed between the applied salt concentration and DPPH for the *P. tricornutum* samples (Figure 1) while a negative and strong correlation (*r* = −0.93) was found for the *S. platensis* samples (Figure 2). As can be seen in Table 1, increasing or decreasing salt concentrations negatively affected the biosynthesis of the phenolic compounds leading to a decrease in the amount of total phenolic content (TPC) and antioxidant capacity obtained by the DPPH method. German-Báez et al. [9] determined the DPPH capacity of the *P. tricornutum* samples as 9.54 mM Trolox/g DW while Kuatrakul et al. [23] reported a DPPH capacity for the *S. platensis* samples as 69.82 mg/100 g DW. Regarding the effect of salt stress on the antioxidant activity in plants, it was reported in some previous studies that higher salt concentrations increased the antioxidant activity by causing abiotic stress [24] while some other studies reported reductions in the antioxidant activity values [25]. In line with previous studies, it is believed that salt stress induces abiotic stress leading to an imbalance between the production and inhibition mechanisms of the reactive oxygen species (ROS) ultimately reducing antioxidant activity.

#### 3.1.2. ABTS Method Results

The highest ABTS capacity was determined as 141.89 and 655.59 mM Trolox/100 g DW in the P30-C and S20-C coded samples, respectively (*p* < 0.05) (Table 1). In a previous study examining the chemical composition and physicochemical properties of *P. tricornutum*, the ABTS capacity was determined as 67.93 mM Trolox/g DW [9]. A positive and moderate correlation (*r* = 0.67) was found for the *P. tricornutum* samples while a negative and strong (*r* = −0.86) correlation was observed for the *S. platensis* samples between the salt concentration and ABTS in the present study. It was reported in a previous study that the maximum antioxidant capacity of the *P. tricornutum* samples evaluated using the ABTS method was found as 758.28 M TE and this value was obtained through extraction for 28.36 min under optimal conditions of 20 °C temperature and 5.5 pH [26]. Goiris et al. [5] reported that the ABTS capacity of *P. tricornutum* ranged between 4.55 and 48.90 µmol Trolox/g DW. It was determined that the antioxidant capacity values obtained in the current study were higher compared to the data of these previous studies.

#### 3.1.3. CUPRAC Method Results

In the present study, the CUPRAC values of the samples exhibited significant differences (*p* < 0.05) as indicated in Table 1. The CUPRAC values ranged from 31.14 to 44.00 to 48.32 to 104.96 mM Trolox/100 g DW in the *P. tricornutum* and *S. platensis* samples, respectively (Table 1). It was observed that the highest CUPRAC values were observed in the control groups for both species. There was a positive and moderate correlation (*r* = 0.23) for the *P. tricornutum* samples while a negative and strong (*r* = −0.83) correlation was observed for the *S. platensis* samples between the applied salt concentrations and CUPRAC values (Figure 1 and Figure 2). It was determined that the antioxidant capacities decreased depending on the applied salt concentrations and *S. platensis* had higher antioxidant activity than *P. tricornutum*. Golmakani et al. [27] reported a CUPRAC value of 78.32 mg ascorbic acid/mL for *S. platensis*. Salt stress causes oxidative stress by disrupting the balance between stimulation and elimination of the reactive oxygen species and excessive radical species such as H_2_O_2_, O_2,_ and OH cause cell death by damaging algal cell components [5].

### 3.2. Total Phenolic Compounds (TPC) Analysis Results

Total phenolic compounds (TPC) of the *P. tricornutum* and *S. platensis* samples are presented in Table 1. The highest TPC was observed in the control samples with 82.46 and 204.80 mg GA/100 g DW in the P30-C and S20-C samples, respectively (*p* < 0.05). There was a positive and moderate (*r* = 0.66) correlation for the *P. tricornutum* samples and a negative and strong (*r* = −0.94) correlation for the *S. platensis* samples between the applied salt concentration and TPC values (Figure 1 and Figure 2). The increase or decrease in the salt amounts in the growing media caused a decrease in the amount of TPC similar to the antioxidant capacity. In three previous studies, the TPC contents of the *S. platensis* samples were reported as 12.2 g/kg by Bolanho et al. [28], 146 mg GA/100 g by Esquivel-Hernández et al. [29], and 318–340 mg GA/100 g by Martelli et al. [30]. Elloumi et al. [31] utilized different amounts of NaCl in an MDM medium to test the influence of salinity on the development and production of *Scenedesmus* sp. microalgae. They determined that high salinity inhibited microalgae growth but low salinity promoted their growth. Furthermore, with low-concentration salt stress, chlorophyll, and carotenoid levels increased. BenMoussa-Dahmen et al. [32] demonstrated that the growth of *Dunaliella* sp. and *Amphora subtropica* was elevated under 3 M NaCl and 1 M NaCl, respectively, and decreased below and above these optimal salt concentrations implying that salinity played a significant role in microalgal growth and even required for the growth of halophilic species such as *Dunaliella* sp. and *A. subtropica*.

In a study examining the effects of different drying processes on the physical properties of the *S. platensis* samples, the TPC content was found to be 371.43 mg GA/100 g [23]. In general, the samples of the *S. platensis* species were found to have higher TPC and antioxidant capacity compared to *P. tricornutum* in the present study. In both species, the control samples had the highest TPC and antioxidant capacity. The TPC and antioxidant capacity, which were found to be low at 15‰ salt concentration (P15) in the *P. tricornutum* samples, increased up to 30‰ (P30-C) salt concentration and then had a decrease. In the *S. platensis* species, the sample grown with the 20‰ control salt amount (S20-C) had the highest TPC and antioxidant capacity but increasing salt concentration caused this value to decrease. When the data obtained in the current study were compared with the data reported in the literature, some variations were observed in the antioxidant capacity results. Microalgae can prevent the effect of reactive oxygen species (ROS) by using antioxidant response mechanisms. Thus, the ROS and antioxidant response mechanism varies according to microalgae species and depend on cell size, cell shape, cell density, growth stage, light, temperature, nutrients, and abiotic stress factors [33]. Other important parameters affecting the amount of phenolic compounds are the extraction conditions. Various factors such as time, temperature, and the type of solvent, can influence the quantity of the phenolic compounds. Optimizing these extraction conditions is essential to maximize the phenolic compound yield. A study identified time as the primary factor in extracting phenolic compounds from *P. tricornutum* cultures. The study found that the total phenolic content increased up to 16 min during the extraction process, after which it started to decrease [26]. These findings align with the results reported by Parniakov et al. [34] for *Nannochloropsis* spp., who demonstrated that the optimal extraction of TPCs using ultrasound assistance was achieved after 15 min.

### 3.3. LC-ESI-MS/MS Phenolic Compounds Analysis Results

**Figure 3:**
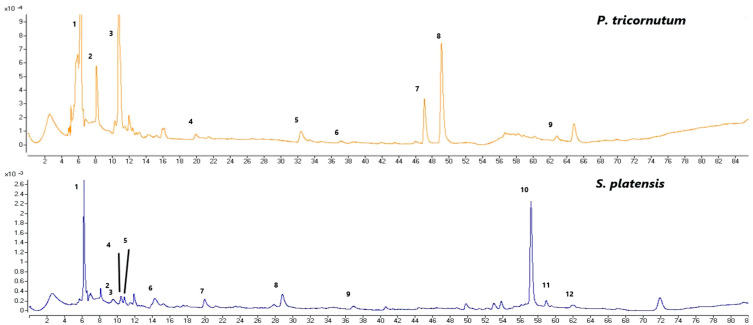
The LC-MS-TIC chromatograms of the phenolic compounds of the *P. tricornutum* samples (**top**); 1: dimethoxyflavone, 2: quinic acid, 3: cinnamic acid, 4: protocatechic acid, 5: vanillic acid, 6: epicatechin, 7: lutein, 8: diatoxanthin, and 9: kaempherol (the peaks correspond to the compounds in Table 2). The LC-DAD-ESI-MS/MS chromatograms of the phenolic compounds from the *S. platensis* samples (**bottom**); 1: catechin derivative, 2: isoferulic acid, 3: o-coumaric acid, 4: caffeic acid derivative, 5: ferulic acid, 6: gallic acid, 7: protocatechic acid, 8: chlorogenic acid, 9: epicatechin, 10: quercetin derivative, 11: quercetin, and 12: kaemferol (the peaks correspond to the compounds in Table 3).

The phenolic compounds identified and quantified in the *P. tricornutum* and *S. platensis* samples grown with varying salt concentrations are given in Table 2 and Table 3, respectively. Then, 20 phenolic compounds were identified and quantified in the *P. tricornutum* samples (Table 2, Figure 3). The amount of these compounds varied between 68 and 96 mg/100 g DW. It was found that the change in the salt concentration significantly decreased the TPC (*p* < 0.05). There was a negative and strong correlation between the applied salt concentrations and quinic acid (*r* = −0.97) and *p*-hydroxybenzoic acid (*r* = −0.97) of the *P. tricornutum* samples (Figure 1) while a positive and moderate correlation was found for the catechin (*r* = 0.54), caffeyl alcohol (*r* = 0.65) and luteolin (*r* = 0.64) (Figure 1). The most dominant phenolic compound was dimethoxyflavone while *trans*-cinnamic acid (LOD- LOQ: 0.08–0.23 µg/mL, *R*^2^: 0.996), 4-hydroxycinnamic acid, cinnamic acid, dihydroxy-dimethoxyflavone, derivative, lutein, and diatoxanthin were also abundant in the *P. tricornutum* samples. In addition, phloroglucinol (LOD-LOQ: 0.32–0.44 µg/mL, *R*^2^: 0.995), protocatechuic acid (LOD-LOQ: 0.02–0.07 µg/mL, *R*^2^: 0.995), *p*-hydroxybenzoic acid (LOD-LOQ: 0.04–0.12 µg/mL, *R*^2^: 0.996), catechin (LOD-LOQ: 0.11–0.37 µg/mL, *R*^2^: 0.995), vanillic acid (LOD-LOQ: 0.06–0.18 µg/mL, *R*^2^: 0.995), caffeic acid, epicatechin (LOD- LOQ: 0.13–0.47 µg/mL, *R*^2^: 0.995), caffeoyl alcohol and derivatives and kaempferol (LOD- LOQ: 0.05–0.17 µg/mL, *R*^2^: 0.995) were determined in the *P. tricornutum* samples. Due to their multiple biological activities, dimethoxyflavone and its derivatives have received great attention recently. They are known to strengthen the TJ barrier (the tight connection between epithelial cells) in intestinal Caco-2 cells. It was observed in the present study that the amount of this compound varied between 20.40 and 31.49 mg/100 g DW and was the highest in the control group. The change in the amount of salt in the growing medium caused a change in the dimethoxyflavone quantity. Cinnamic acid (LOD- LOQ: 0.06–0.18 µg/mL, *R*^2^: 0.995) and caffeic acid (LOD-LOQ: 0.02, 0.04 µg/mL, *R*^2^: 0.996) are within the hydroxycinnamic acid group containing nine carbon atoms [35] and their amounts varied from 6.51 to 8.83 mg/100 g in the current study. It was reported in many studies that cinnamic acid and caffeic acid have anticancer, antioxidant, antibacterial, anti-inflammatory, and antidiabetic activities [35].

Lutein (LOD-LOQ: 0.08–0.28 µg/mL, *R*^2^: 0.995), known as the carotenoid vitamin and having covalent bonds with fatty acids, is a yellow-colored organic compound available in many organisms including plants, bacteria, algae, yeasts, plants, etc. [36].

Microalgae have become a potential alternative to the carotenoid due to their high lutein content and biomass productivity [37]. It was observed in the present study that the amount of lutein varied between 4.03 and 5.21 mg/100 g and was higher in the control groups and decreased depending on the salt concentration. Diatoxanthin on the other hand, is a xanthophyll species found in phytoplankton and diatoms and its amount was determined as 4.43–6.19 mg/100 g. This compound has great importance for the food, cosmetic, and pharmaceutical industries due to its beneficial activities such as antioxidant, anticancer, anti-inflammatory, anti-obesity, and neuroprotective [7].

In the *S. platensis* samples, 24 phenolic compounds were identified and quantified (Table 3). Their concentrations varied between 73 and 124 mg/100 g DW and their amount decreased significantly (*p* < 0.05) depending on the increasing salt concentration. There was a positive and moderate (*r* = 0.44) correlation between the applied salt concentrations and catechin derivative compounds of the *S. platensis* samples (Figure 2) and a negative and strong correlation was observed for the other compounds. It was observed that the dominant phenolic compound was quercetin-derived in the *S. platensis* samples while gallic acid (LOD-LOQ: 1.89–6.30 µg/mL, *R*^2^: 0.995), catechin-derived compound, isoferulic acid (LOD-LOQ: 0.20–0.60 µg/mL, *R*^2^: 0.995), p-hydroxybenzoic acid(LOD-LOQ: 0.04–0.12 µg/mL, *R*^2^: 0.996), protocatechuic acid (LOD-LOQ: 0.02–0.07 µg/mL, *R*^2^: 0.995), catechin (LOD-LOQ: 0.11–0.37 µg/mL, *R*^2^: 0.995), vanillic acid (LOD-LOQ: 0.06–0.18 µg/mL, *R*^2^: 0.995), epicatechin (LOD-LOQ: 0.13–0.42 µg/mL, *R*^2^: 0.995) were also abundant. In addition, 5,7-dihydroxy-3′,4′-dimethoxyflavanone, *o*-coumaric acid (LOD-LOQ: 0.24–0.82 µg/mL, *R*^2^: 0.995), 4-hydroxycinnamic acid, caffeic acid (LOD-LOQ: 0.01–0.04 µg/m, *R*^2^: 0.996) and derivatives, ferulic acid (LOD-LOQ: 0.18–0.60 µg/m, *R*^2^: 0.995), chlorogenic acid(LOD-LOQ: 0.02–0.07 µg/mL, *R*^2^: 0.995), caffeic acid, epicatechin, phloroglucinol(LOD-LOQ: 0.32–0.44 µg/mL, *R*^2^: 0.995), lutein(LOD-LOQ: 0.08–0.28 µg/mL, *R*^2^: 0.995) carotenoid derivative, quercetin and kaempferol (LOD-LOQ: 0.05–0.17 µg/mL, *R*^2^: 0.995)were also quantified in the *S. platensis* samples. It was seen that the amount of quercetin varied between 13 and 18 mg/100 g DW and was higher in the S25 and S30 coded samples. This compound is a plant flavonol from the flavonoid group of polyphenols commonly found in nature. It is a powerful antioxidant with anti-inflammatory, antihypertensive, anti-obesity, antihypercholesterolemic, and antiatherosclerotic activities [38]. The amount of gallic acid in the *S. platensis* samples varied between 8.44 and 11.13 mg/100 g while its highest content was detected in the control sample (S20-C) and increasing salt content caused a decrease in its amount in the current study. Gallic acid or 3,4,5-trihydroxy benzoic acid is one of the most abundant phenolic acids in plants with a colorless or slightly yellow crystalline structure and has wide applications in the food and pharmaceutical industries with therapeutic activities in gastrointestinal, neuropsychological, metabolic, and cardiovascular disorders due to its antioxidant, anti-inflammatory and antineoplastic properties [39]. It was also found in the present study that the amount of catechin, a flavonoid group compound, varied between 2.59 and 6.29 mg/100 g and the amount of epicatechin changed from 2.53 to 8.73 mg/100 g. These compounds were at the highest amounts in the S20-C coded sample and increasing salt concentration led to a significant decrease in their quantities.

Catechins are available in plants and are important secondary metabolites with high antioxidant potential [29]. It was observed in the current study that the amount of vanillic acid (4-hydroxy-3-methoxybenzoic acid) was between 3.03 and 7.43 mg/100 g and decreased with increasing salt concentration. Vanillic acid is a metabolic byproduct of caffeic acid and has significant benefits with its antioxidant, anticancer, anti-obesity, antidiabetic, antibacterial, and anti-inflammatory effects. The amount of phloroglucinol and kaempferol varied from 1.96 to 2.40 mg/100 g and from 0.17 to 0.75 mg/100 g, respectively, and increasing salt concentration caused a reduction in their amounts. The quantity of chlorogenic acid varied between 1.06 and 2.99 mg/100 g. As the phenolic compounds in the *S. platensis* samples, *p*-hydroxybenzoic acid, protocatechuic acid, vanillic acid, gallic acid, syringic acid, 4-hydroxybenzaldehyde, 3,4-dihydroxy benzaldehyde, *o*- and *p*-coumaric acid, caffeic acid, ferulic acid, sinapic acid and chlorogenic acid were determined [4,5,6]. It was generally observed that the *S. platensis* samples had a higher phenolic potential compared to the *P. tricornutum* samples but there were reductions in their amounts depending on the increasing salt concentration (Table 2 and Table 3). Regarding the correlation analysis, a strong correlation was observed between the salt concentration and the phenolic compounds of the *P. tricornutum* and *S. platensis* samples (Figure 1 and Figure 2).

### 3.4. Results of the Bioaccessibility of the Bioactive Compounds by the In Vitro Digestion

The bioaccessibility of the polyphenols from freeze-dried *P. tricornutum* and *S. platensis* extract samples was assessed using a three-stage in vitro gastrointestinal digestion model [21] that mimicked oral, gastric, and intestinal digestion processes. The antioxidant activities and TPCs of the upper phase samples obtained from this model are presented in Table 4. Significant differences were observed between the oral, gastric, and intestinal samples (*p* < 0.05).

Regarding the *P. tricornutum* samples, the highest DPPH amounts were determined as 0.05, 0.31, and 0.45 mM Trolox/100 g DW while the highest ABTS amounts were 0.35, 0.72, and 16.96 mM Trolox/100 g DW and the highest CUPRAC quantities were 64.23, 62.90 and 1151.18 mM Trolox/100 g DW in the mouth, stomach, and intestines in the P30-C control sample, respectively (Table 4). The TPC amounts were determined as 69.22, 517.98, and 557.97 mg/100 g DW, respectively. The order of the DPPH, ABTS, CUPRAC, and TPC amounts of the *P. tricornutum* samples was “intestinal > gastric > oral”. The lowest amount of DPPH in the mouth was determined as 0.03 mM Trolox/100 g DW in P15, P25, and P35 coded samples, and 0.15 and 0.18 mM Trolox/100 g DW in the P35 sample in the stomach and intestines, respectively. The amounts of the ABTS in the mouth were determined as 0.27 mM Trolox/100 g DW in the P15 sample and 0.63 and 13.89 mM Trolox/100 g DW in the P35 sample in the stomach and intestines, respectively. The lowest amounts of CUPRAC were calculated as 32.20, 43.50, and 649.06 mM Trolox/100 g in the mouth, stomach and intestine sample, respectively, and the TPC amounts were calculated as 53.93 mg/100 g in the P25 coded sample in the mouth, 130.07 mg/100 g in the P15 sample in the stomach and 491.14 mg/100 g in the intestinal P35 sample.

For the *S. platensis* samples, the highest DPPH amounts were determined in the mouth, stomach, and intestines as 0.09, 0.40, and 0.87 mM Trolox/100 g DW in the S20-C control sample, and the ABTS values were 5.26, 5.79 and 37.26 mM Trolox/100 g DW while the CUPRAC quantities were 78.58, 86.83 and 7078.48 mM Trolox/100 g DW and the TPC amounts were determined as 717.38, 1325.05 and 1641.55 mg/100 g DW, respectively (Table 4). The DPPH, ABTS, CUPRAC, and TPC amounts of the *S. platensis* samples were observed in the order of “intestinal > gastric > oral”. The lowest amounts of DPPH in the mouth, stomach, and intestines were determined as 0.05, 0.23, and 0.68 mM Trolox/100 g DW in the S30 and S35 samples, respectively. The amounts of the ABTS in the mouth, stomach, and intestines were determined as 3.98, 4.66, and 22.95 mM Trolox/100 g DW in the S30 sample, respectively. The lowest amounts of the CUPRAC were calculated as 62.49, 69.70, and 4155.43 mM Trolox/100 g in the S35 sample in the mouth, stomach, and intestines, respectively, while the TPC values were calculated as 554.70, 904.60 and 1139.18 mg/100 g in the S30 sample in the mouth, stomach, and intestines, respectively. The total concentration of a compound in food can significantly differ from the actual amount that is biologically accessible. Thus, understanding the changes and the bioaccessibility occurring during digestion is necessary for estimating bioaccessibility and bioactivity [40].

*P. tricornutum* species has a ciliated cell wall while *S. platensis* species has a non-cellulosic and 86% digestible cell wall. The integrity of cell walls can significantly limit the presence and activity of compounds such as vitamins, pigments, and fatty acids; thus, the disruption of the microalgal cell wall is required as a pretreatment to allow the release of the cellular contents [41]. It was determined in the present study that the antioxidant capacity and TPC amounts of the *S. platensis* samples in the mouth, stomach, and intestinal phases were higher than those of the *P. tricornutum* samples (Table 4). This may be attributed to the fact that *S. platensis* has a non-cellulosic and easily degradable cell wall compared to *P. tricornutum*. Algal proteins and carbohydrates that are not fully digested in the small intestine can benefit the gastrointestinal system by indirectly stimulating the immune response by supporting microbial responses [42].

### 3.5. Microbiological Analysis Results

Microbiological analyses are applied to foods mainly to reveal the presence of unwanted microorganisms and determine the food’s suitability for human consumption [43]. The total number of aerophilic and mesophilic bacteria gives information about the possible shelf life of the food and the contamination levels of the food in the production stages [44]. If the total number of aerobic mesophilic microorganisms, which is used as an indicator in the determination of general hygiene and microbial load, is high, then the amount of other microbial groups will also be high [44].

The total amounts of aerobic mesophilic bacteria and yeast/mold count of the *P. tricornutum* and *S. platensis* samples obtained in the current study are given in Table 5. The total numbers of aerobic mesophilic bacteria in the *P. tricornutum* and *S. platensis* samples cultured by adding different salt concentrations to the growing medium were found to be 300–2.78 × 10^4^ cfu/g for the *P. tricornutum* samples and 300–1.9 × 10^4^ cfu/g for the *S. platensis* samples. In general, if the total number of aerobic mesophilic bacteria is over 10^5^ cfu/g in a food sample, it is an indication that general hygiene rules are not followed during the preparation of that food. Hence, it was determined that the total number of aerobic mesophilic bacteria obtained in the present study was at an acceptable level. According to the European Union (EU) standards, the critical level for the total number of aerobic mesophilic bacteria is 10^5^ cfu/mL while it is 10^3^ cfu/g-ml according to the Turkish food codex (TFC) [45].

The yeast/mold counts of the *P. tricornutum* and *S. platensis* samples were found to be 10–1.35 × 10^4^ cfu/g for the *P. tricornutum* samples and 10–1.0 × 10^4^ cfu/g for the *S. platensis* samples (Table 5). The critical acceptability level for the yeast/mold count is 10^4^ according to the EU while it is 10^3^ cfu/mL according to the TFC and the World Health Organization (WHO) standards. The data obtained from the current study were found to be moderately acceptable according to the EU, WHO, and TFC standards [45]. In a study conducted with *Spirulina* grown in Morocco, the total number of aerobic mesophilic bacteria was found to be 208 cfu/mL and the yeast and mold counts were quantified as 14 cfu/mL [6].

## 4. Conclusions

The effects of different salt concentrations of the growth medium on the bioactive compounds, antioxidant activities, and in vitro bioaccessibility of two different microalgae (*P. tricornutum* and *S. platensis*) were investigated in this study. The highest antioxidant capacity (AC) and total phenolic substances (TPC) were determined in the P30-C and S20-C control groups. Then, 20 and 24 phenolic compounds (PC) were identified and quantified by LC-ESI-MS/MS in the *P. tricornutum* and *S. platensis* samples, respectively. It was observed that the increase in the salt concentration decreased the amount of TPC. The dominant PC was dimethoxyflavone while trans-cinnamic acid, 4-hydroxycinnamic acid, cinnamic acid, dihydroxy-dimethoxyflavone derivative, lutein, and diatoxanthin were abundant in the *P. tricornutum* samples. In the *S. platensis* samples, on the other hand, the dominant PC was quercetin derivative while gallic acid, catechin derivative, isoferulic acid, *p*-hydroxybenzoic acid, protocatechuic acid, catechin, vanillic acid, and epicatechin were abundant. The changes in the AC and TPC in the upper phase samples obtained from the three-stage in vitro digestion model, including mouth, stomach, and intestine were examined and the highest values were observed in the order of “intestine > stomach > mouth” phases in P30-C and S20-C control samples, respectively. The total number of aerobic mesophilic bacteria was determined to be 300–2.78 × 10^4^ cfu/g for the *P. tricornutum* samples and 300–1.9 × 10^4^ cfu/g for the *S. platensis* samples that were obtained by adding various salt concentrations to the growing medium. Generally, if there are more than 105 cfu/g of aerobic mesophilic bacteria in a food sample, it means that general hygiene standards were not observed during the item’s preparation. Hence, it was determined that the total number of aerobic mesophilic bacteria obtained in the present study was at an acceptable level. In sum, both species studied in this work were found to be rich in terms of bioactive substances but the solubility of these compounds was not sufficient; thus, innovative extraction techniques should be included in future studies.

## Figures and Tables

**Table 1:** Results of the antioxidant capacity (DPPH, ABTS, and CUPRAC) and total phenolic compounds (TPC) in the freeze-dried *P. tricornutum* and *S. platensis* powder samples depending on the growing medium salt concentration.

Species	Salt Concentration	Analysis *			
		DPPH	ABTS+	CUPRAC	TPC
*P. tricornutum*	P15	27.47 ± 0.83 ^Aa^	97.65 ± 3.21 ^Aa^	32.12 ± 0.27 ^Aa^	63.51 ± 0.43 ^Aa^
	P25	56.80 ± 2.09 ^Bb^	123.18 ± 0.40 ^BCbc^	32.02 ± 0.45 ^Aa^	75.95 ± 1.37 ^Bb^
	P30-C	79.40 ± 1.71 ^Cc^	141.89 ± 2.85 ^Cc^	44.00 ± 0.69 ^Bb^	82.46 ± 1.07 ^Cc^
	P35	29.76 ± 0.50 ^Aa^	119.05 ± 2.61 ^Bb^	31.14 ± 0.09 ^Aa^	72.63 ± 0.30 ^Bb^
*S. platensis*	S20-C	172.67 ± 3.21 ^Fc^	655.59 ± 12.05 ^Gd^	104.96 ± 2.27 ^Fd^	204.80 ± 0.66 ^Fc^
	S25	151.65 ± 2.65 ^Eb^	425.43 ± 12.59 ^Fc^	48.32 ± 0.04 ^Ca^	171.18 ± 0.96 ^Eb^
	S30	140.66 ± 0.96 ^Da^	404.30 ± 1.39 ^Eb^	77.03 ± 1.61 ^Ec^	166.00 ± 0.49 ^Da^
	S35	137.18 ± 0.50 ^Da^	373.78 ± 3.11 ^Da^	70.77 ± 1.76 ^Db^	163.96 ± 2.84 ^Da^

DPPH, ABTS, and CUPRAC: mM Trolox/100 g DW, TPC: mg GA/100 g DW; DW: Dry weight; Different superscripts in the same column indicate statistical differences at *p* < 0.05. * (a–d): indicates the statistical differences within the salt concentrations separately for the *P. tricornutum* and *S. platensis* samples. * (A–G): indicates the statistical differences between the *P. tricornutum* and *S. platensis* samples.

**Table 2:** Phenolic compounds and their amounts in the freeze-dried *P. tricornutum* samples (mg/100 g DW) depending on the growing medium salt concentration.

No	RT (min)	Phenolic Compounds	^x^[M−H]^−^/^y^[M+H]^+^	MS^2^	P15	P25	P30-C	P35
1	6.08	Catechin isomer	289 ^x^	267/245/172/154	7.19 ± 0.17 ^b^	10.48 ± 0.35 ^d^	8.09 ± 0.07 ^c^	5.54 ± 0.43 ^a^
2	6.31	Dimethoxyflavone	281 ^x^	267	29.90 ± 0.49 ^b^	31.36 ± 0.22 ^c^	31.49 ± 0.23 ^c^	20.40 ± 0.28 ^a^
3	8.10	Quinic acid	190 ^x^	85	7.75 ± 0.12 ^c^	5.95 ± 0.07 ^b^	5.38 ± 0.08 ^a^	5.22 ± 0.14 ^a^
4	10.61	Trans-cinnamic acid	147 ^x^	103	3.15 ± 0.13 ^c^	1.72 ± 0.05 ^a^	4.31 ± 0.10 ^d^	2.33 ± 0.07 ^b^
5	10.64	4-hydroxycinnamic acid	163 ^x^	145/141/119	5.00 ± 0.02 ^c^	3.72 ± 0.17 ^b^	5.56 ± 0.05 ^d^	2.90 ± 0.07 ^a^
6	10.72	Cinnamic acid	147 ^x^	103	8.44 ± 0.26 ^c^	6.51 ± 0.00 ^a^	8.83 ± 0.14 ^c^	7.76 ± 0.29 ^b^
7	11.88	Quercetin-3-O-rutinoside	609 ^x^	300	1.27 ± 0.01 ^a^	2.10 ± 0.10 ^c^	1.70 ± 0.03 ^b^	1.51 ± 0.09 ^b^
8	19.26	Phloroglucinol	127 ^y^	108	0.42 ± 0.00 ^b^	0.34 ± 0.00 ^a^	0.58 ± 0.03 ^c^	0.31 ± 0.01 ^a^
9	20.03	Protocatechuic acid	153 ^x^	109	0.52 ± 0.02 ^a^	0.70 ± 0.00 ^b^	0.91 ± 0.05 ^c^	0.47 ± 0.01 ^a^
10	26.78	*p*-hydroxybenzoic acid	137 ^x^	109/93	1.12 ± 0.03 ^c^	0.54 ± 0.02 ^b^	0.51 ± 0.05 ^b^	0.20 ± 0.02 ^a^
11	27.99	Catechin	289 ^x^	245	0.20 ± 0.02 ^a^	0.27 ± 0.04 ^ab^	0.53 ± 0.00 ^c^	0.30 ± 0.04 ^b^
12	28.50	Dihydroxy-dimethoxyflavone derivative	607 ^x^	315	6.36 ± 0.00 ^a^	8.15 ± 0.14 ^c^	9.92 ± 0.02 ^d^	7.09 ± 0.14 ^b^
13	33.59	Vanillic acid	167 ^x^	122	0.30 ± 0.05 ^b^	0.27 ± 0.01 ^ab^	0.40 ± 0.02 ^c^	0.20 ± 0.02 ^a^
14	34.71	Caffeic acid	179 ^x^	135	0.29 ± 0.01 ^b^	0.17 ± 0.05 ^a^	0.34 ± 0.00 ^b^	0.17 ± 0.01 ^a^
15	37.16	Epicatechin	289 ^x^	245	1.11 ± 0.03 ^c^	0.72 ± 0.00 ^b^	1.72 ± 0.01 ^d^	0.59 ± 0.01 ^a^
16	43.95	Caffeyl alcohol	164 ^x^	145/121/103	2.16 ± 0.00 ^a^	2.36 ± 0.05 ^b^	3.24 ± 0.05 ^d^	2.62 ± 0.08 ^c^
17	45.01	Caffeyl alcohol derivative	164 ^x^	103	0.24 ± 0.02 ^a^	0.58 ± 0.07 ^c^	0.86 ± 0.01 ^d^	0.35 ± 0.03 ^b^
18	47.17	Lutein	569 ^y^	551/533/578/495/119/145/121	4.03 ± 0.05 ^a^	4.39 ± 0.23 ^ab^	5.21 ± 0.15 ^c^	4.52 ± 0.02 ^b^
19	50.19	Diatoxanthin	566 ^y^	331/341/360	4.92 ± 0.14 ^b^	4.43 ± 0.14 ^a^	6.19 ± 0.07 ^d^	5.40 ± 0.08 ^c^
20	62.89	Kaempferol	285 ^x^	257/229/216	0.56 ± 0.00 ^b^	1.49 ± 0.20 ^c^	0.43 ± 0.00 ^a^	0.40 ± 0.02 ^a^
		**Total**			**84.93 ± 1.32 ^b^**	**86.25 ± 1.91 ^c^**	**96.99 ± 1.16 ^d^**	**68.28 ± 1.54 ^a^**

DW: Dry weight; RT: Retention time. Different letters (a–d) on the same row indicate statistical differences (*p* < 0.05)**.** x: Negative ionization mode. y: Positive ionization mode.

**Table 3:** Phenolic compounds and their amounts in the freeze-dried *S. platensis* samples (mg/100 g DW) depending on the growing medium salt concentration.

No	RT (min)	Compounds	^x^[M−H]^−^/^y^[M+H]^+^	MS^2^	S20-C	S25	S30	S35
1	5.53	Catechin derivative	289 ^x^	245	8.07 ± 0.06 ^b^	7.22 ± 0.20 ^a^	7.17 ± 0.03 ^a^	9.16 ± 0.17 ^c^
2	7.97	5,7-Dihydroxy-3′,4′-dimethoxyflavanone	315 ^x^	283/245/215/195	1.34 ± 0.07 ^c^	0.84 ± 0.02 ^b^	0.57 ± 0.08 ^a^	0.42 ± 0.06 ^a^
3	9.56	Isoferulic acid	195 ^x^	178/133/121	9.33 ± 0.08 ^d^	4.90 ± 0.04 ^b^	8.28 ± 0.02 ^c^	3.67 ± 0.12 ^a^
4	10.53	*o*-coumaric acid	165 ^y^	147/123	5.67 ± 0.03 ^c^	1.17 ± 0.03 ^b^	0.69 ± 0.15 ^a^	0.60 ± 0.12 ^a^
5	10.64	4-Hydroxycinnamic acid	163 ^x^	145/141/119	3.33 ± 0.02 ^c^	2.21 ± 0.08 ^b^	2.99 ± 0.25 ^c^	1.57 ± 0.17 ^a^
6	10.88	Caffeic acid derivative	179 ^x^	135	4.12 ± 0.30 ^b^	1.74 ± 0.62 ^a^	1.95 ± 0.04 ^a^	1.70 ± 0.02 ^a^
7	11.49	Ferulic acid	195 ^y^	177/145	2.35 ± 0.06 ^c^	0.65 ± 0.06 ^a^	0.84 ± 0.11 ^ab^	0.92 ± 0.02 ^b^
8	11.93	7/8-Dihydroxycoumarin	178 ^y^	117/109	6.47 ± 0.16 ^d^	2.23 ± 0.00 ^c^	0.50 ± 0.00 ^a^	0.86 ± 0.00 ^b^
9	14.09	Gallic acid	169 ^x^	125	11.13 ± 0.11 ^c^	9.95 ± 0.12 ^b^	8.44 ± 0.03 ^a^	9.80 ± 0.30 ^b^
10	15.2	6-Methox-7-hydroxycoumarin	191 ^x^	177/162/103	4.05 ± 0.53 ^b^	2.14 ± 0.14 ^a^	1.76 ± 0.11 ^a^	3.33 ± 0.22 ^b^
11	19.96	Phloroglucinol	127 ^y^	108	2.40 ± 0.01 ^b^	2.22 ± 0.07 ^ab^	1.96 ± 0.05 ^a^	2.02 ± 0.20 ^a^
12	20.09	Protocatechuic acid	153 ^x^	109	3.44 ± 0.10 ^c^	2.66 ± 0.10 ^b^	2.68 ± 0.14 ^b^	2.00 ± 0.03 ^a^
13	23.36	Catechin gallate	441 ^x^	291/245/220/195/160	4.59 ± 0.72 ^b^	6.57 ± 0.21 ^c^	1.74 ± 0.26 ^a^	2.00 ± 0.35 ^a^
14	26.78	*p*-hydroxybenzoic acid	137 ^x^	109/93	5.97 ± 0.07 ^b^	1.80 ± 0.41 ^a^	2.96 ± 0.86 ^a^	2.68 ± 0.12 ^a^
15	27.88	Catechin	289 ^x^	245	6.29 ± 0.03 ^c^	3.97 ± 0.00 ^b^	3.39 ± 0.41 ^b^	2.59 ± 0.16 ^a^
16	28.00	Chlorogenic acid	353 ^x^	191	2.99 ± 0.04 ^b^	1.23 ± 0.02 ^a^	1.10 ± 0.03 ^a^	1.06 ± 0.13 ^a^
17	34.60	Vanillic acid	167 ^x^	122	7.43 ± 0.13 ^c^	6.19 ± 0.40 ^b^	5.47 ± 0.11 ^b^	3.03 ± 0.68 ^a^
18	34.70	Caffeic acid	179 ^x^	135	1.48 ± 0.05 ^c^	0.92 ± 0.03 ^a^	1.31 ± 0.00 ^b^	1.23 ± 0.05 ^b^
19	36.00	Epicatechin	289 ^x^	245	8.73 ± 0.90 ^b^	3.22 ± 0.94 ^ab^	2.53 ± 0.47 ^a^	5.55 ± 0.83 ^ab^
20	47.17	Lutein	569 ^y^	551/533/578/495/119/145/121	2.18 ± 0.05 ^c^	0.54 ± 0.05 ^a^	0.63 ± 0.06 ^a^	1.02 ± 0.04 ^b^
21	47.38	Carotenoid derivative	566 ^y^	109	1.46 ± 0.02 ^c^	1.23 ± 0.03 ^b^	0.87 ± 0.02 ^a^	0.84 ± 0.00 ^a^
22	57.18	Quercetin derivative	303 ^y^	257/285	16.19 ± 0.13 ^b^	18.08 ± 0.18 ^c^	18.08 ± 0.35 ^c^	13.38 ± 0.07 ^a^
23	61.00	Quercetin	549 ^x^	463/301/161	5.11 ± 0.05 ^d^	2.79 ± 0.00 ^a^	3.32 ± 0.03 ^b^	4.06 ± 0.14 ^c^
24	62.89	Kaempferol	285 ^x^	257/229/216	0.75 ± 0.04 ^c^	0.33 ± 0.00 ^b^	0.66 ± 0.04 ^c^	0.17 ± 0.00 ^a^
		**Total**			**124.87 ± 3.64 ^d^**	**84.8 ± 3.75 ^c^**	**79.89 ± 3.65 ^b^**	**73.63 ± 4.00 ^a^**

DW: Dry weight. RT: Retention time. Different letters (a–d) on the same row indicate statistical differences (*p* < 0.05). x: Negative ionization mode. y: Positive ionization mode.

**Table 4:** The effect of the in vitro digestion model on the antioxidant activity (DPPH, ABTS, and CUPRAC) and total phenolic compounds (TPC) in the freeze-dried *P. tricornutum* and *S. platensis* powder samples depending on the growing medium salt concentration.

		Oral Phase				Gastric Phase				Intestinal Phase			
Species	Salt Concentration	DPPH	ABTS^+^	CUPRAC	TPC	DPPH	ABTS+	CUPRAC	TPC	DPPH	ABTS+	CUPRAC	TPC
*P. tricornutum*	P15	0.03 ± 0.00 ^Aa^	0.27 ± 0.01 ^Aa^	42.39 ± 1.97 ^Bb^	58.13 ± 0.80 ^Aa^	0.21 ± 0.01 ^Bb^	0.73 ± 0.01 ^Aa^	50.74 ± 0.79 ^Bb^	130.07 ± 3.30 ^Aa^	0.31 ± 0.00 ^Bb^	14.77 ± 0.24 ^Bb^	698.23 ± 10.25 ^Aa^	497.82 ± 4.02 ^Bb^
	P25	0.03 ± 0.00 ^Aa^	0.28 ± 0.00 ^Aa^	47.22 ± 0.60 ^Cc^	53.93 ± 3.58 ^Aa^	0.24 ± 0.01 ^Bb^	0.72 ± 0.07 ^Aa^	56.85 ± 0.21 ^Cc^	151.94 ± 4.29 ^Aa^	0.36 ± 0.00 ^Cc^	15.27 ± 0.19 ^Cc^	714.82 ± 14.67 ^Bb^	519.28 ± 1.31 ^Bb^
	P30-C	0.05 ± 0.00 ^Bb^	0.35 ± 0.01 ^Aa^	64.23 ± 0.77 ^Dd^	69.22 ± 0.58 ^Bb^	0.31 ± 0.00 ^Cc^	0.74 ± 0.01 ^Aa^	62.90 ± 1.19 ^Dd^	517.98 ± 1.57 ^Bb^	0.45 ± 0.00 ^Dd^	16.96 ± 0.11 ^Dd^	1151.18 ± 15.20 ^Cc^	557.97 ± 0.90 ^Cc^
	P35	0.03 ± 0.00 ^Aa^	0.29 ± 0.01 ^Aa^	32.20 ± 2.10 ^Aa^	64.78 ± 1.09 ^Aa^	0.15 ± 0.00 ^Aa^	0.63 ± 0.01 ^Aa^	43.50 ± 1.10 ^Aa^	161.94 ± 3.21 ^Aa^	0.18 ± 0.00 ^Aa^	13.89 ± 0.19 ^Aa^	649.06 ± 4.90 ^Aa^	491.14 ± 7.78 ^Aa^
*S. platensis*	S20-C	0.09 ± 0.00 ^Cb^	5.26 ± 0.05 ^Dc^	78.58 ± 1.34 ^Eb^	717.38 ± 4.08 ^Fd^	0.40 ± 0.00 ^Db^	5.79 ± 0.11 ^Cb^	86.83 ± 0.52 ^Eb^	1325.05 ± 25.79 ^Fd^	0.87 ± 0.00 ^Fb^	37.26 ± 0.38 ^Gc^	7078.48 ± 25.24 ^Fc^	1641.55 ± 17.65 ^Fc^
	S25	0.05 ± 0.00 ^Ba^	3.98 ± 0.03 ^Ba^	64.90 ± 0.44 ^Da^	554.70 ± 10.27 ^Ca^	0.23 ± 0.00 ^Ba^	4.66 ± 0.08 ^Ba^	71.72 ± 0.40 ^Da^	904.60 ± 9.43 ^Ca^	0.68 ± 0.01 ^Ea^	22.95 ± 0.38 ^Ea^	4695.91 ± 20.58 ^Eb^	1139.18 ± 7.40 ^Da^
	S30	0.05 ± 0.00 ^Ba^	4.09 ± 0.06 ^Cb^	63.69 ± 0.09 ^Da^	611.4 ± 11.95 ^Db^	0.23 ± 0.00 ^Ba^	4.72 ± 0.17 ^Ba^	70.71 ± 0.32 ^Da^	957.50 ± 11.65 ^Db^	0.68 ± 0.01 ^Ea^	24.09 ± 0.65 ^Fb^	4425.67 ± 16.25 ^Da^	1197.15 ± 3.50 ^Da^
	S35	0.05 ± 0.00 ^Ba^	4.21 ± 0.09 ^Cb^	62.49 ± 1.36 ^Da^	668.10 ± 13.48 ^Ec^	0.23 ± 0.00 ^Ba^	4.79 ± 0.26 ^Ba^	69.70 ± 0.23 ^Da^	1010.41 ± 14.56 ^Ec^	0.68 ± 0.01 ^Ea^	25.24 ± 0.86 ^Fb^	4155.43 ± 11.77 ^Da^	1255.12 ± 4.88 ^Eb^

Different superscripts in the same column indicate statistical differences at *p* < 0.05. (a–d): indicates the statistical differences within the salt concentrations separately in the *P. tricornutum* and *S. platensis* samples. (A–G): indicates the statistical differences between the *P. tricornutum* and *S. platensis* samples.

**Table 5:** The total aerobic mesophilic bacteria and yeast/mold counts of the freeze-dried *P. tricornutum* and *S. platensis* powder samples depending on the growing medium salt concentration.

Species	Salt Concentration	Yeast/Mold Count (cfu/g)	Total Aerobic Mesophilic Bacteria Count (cfu/g)
*P. tricornutum*	P15	<10	2.24 × 10^4^
	P25	1.15 × 10^4^	1.88 × 10^4^
	P30-C	1.25 × 10^4^	>300
	P35	1.35 × 10^4^	2.78 × 10^4^
*S. platensis*	S20-C	<10	>300
	S25	1 × 10^4^	1.7 × 10^4^
	S30	<10	1.9 × 10^4^
	S35	<10	1.21 × 10^4^

## Data Availability

The datasets generated for this study are available on request to the corresponding author.
